# Internet-Delivered Cognitive Behavioral Therapy for Social Anxiety Disorder: Nationwide Retrospective Cohort Observational Study

**DOI:** 10.2196/85676

**Published:** 2026-09-10

**Authors:** Anna Paula Laizane, Ville Ritola, Tom H Rosenström, Suoma Eeva Saarni, Maya L Rutan, Boris Karpov, Satu Pihlaja, Jan-Henry Stenberg, Grigori Joffe

**Affiliations:** 1 Riga Stradiņš University and National Center for Mental Health, Riga, Latvia; 2 Department of Psychiatry, Helsinki University Hospital, Helsinki, Finland; 3 Department of Psychiatry, Faculty of Medicine, University of Helsinki, P.O. Box 3 (Fabianinkatu 33), Helsinki, Uusimaa, 00014, Finland, 358 40 5136500; 4 Faculty of Medicine and Health Technology, University of Tampere, , Tampere, Finland; 5 Family and Social Services, Psychiatry, Well Being Services County of Päijät-Häme, Lahti, Finland; 6 Undergraduate Program in Neuroscience and Faculty of Computing & Data Sciences, Boston University, Boston, MA, United States

**Keywords:** iCBT, cognitive behavioral therapy, routine care, social anxiety disorder, internet, web-based, digital health, mental health

## Abstract

**Background:**

Internet-delivered cognitive behavioral therapies (iCBTs) can address the accessibility and affordability limitations of conventional therapy. Although iCBTs for social anxiety disorder (SAD) are efficacious, their effectiveness in routine care remains less well established. Helsinki University Hospital (HUS) provides a novel nationwide iCBT program for SAD.

**Objective:**

We aimed to assess whether this 7-session, flexible time-scheduled, therapist-supported HUS-iCBT for SAD, delivered by a specialized clinic, (1) is effective in routine care and (2) can enhance adherence, compared to previously published programs with ≥9 sessions.

**Methods:**

This retrospective, registry-based, observational cohort study used data from the HUS-iCBT register from 2019 to 2022. For treatment, the inclusion criteria were a diagnosis of SAD and possession of an email address and an official e-identification. Severe personality disorders, suicidal behavior, acute psychosis or mania, and disorders with cognitive decline were exclusion criteria. Altogether, 2360 physician-referred patients were accepted for therapy. For the study, we applied an additional criterion of ≥21 baseline score on the Social Phobia Inventory (SPIN, primary outcome) to verify the exclusion of subclinical conditions. Patients aged less than 18 years, with missing baseline information, who did not log-in, or reentered treatment were excluded. The remaining patients (n=1654) formed the modified intent-to-treat (mITT) population, provided they completed ≥1 on-therapy sessions. Secondary measures were Overall Anxiety Severity and Impairment Scale and Patient Health Questionnaire-9.

**Results:**

Linear mixed effects modeling revealed a large SPIN-measured effect size for mITT and completer populations (Cohen *d*=1.09, 95% CI 1.03‐1.16 and *d*=1.15, 95% CI 1.06‐1.23, respectively). Late and early dropouts also benefited from the treatment, with medium and small effect sizes, respectively (*d*=0.41, 95% CI 0.32‐0.50 and *d*=0.72, 95% CI 0.58‐0.86). Reliable Change Index analysis showed improvement in 679 out of 1654 (41.1%, 95% CI 38.7‐43.5) patients in the mITT population and 516 out of 893 (57.8%, 95% CI 54.5‐61.0) patients in the completer population. Completers exhibited improvement also on secondary measures. Patients completed a mean of 5.74 (SD 1.99; 82%) of all 7 sessions (vs 59% and 68% in earlier programs with ≥9 sessions and 85.3% in one 6-session program). In the mITT population, 893 out of 1654 (54%, 95% CI 51.6‐56.4) patients were completers. Independent sample *t* test was applied for comparisons between groups. Missing data were analyzed with the Little MCAR (missing completely at random) test. Multiple testing was compensated using the Benjamini-Hochberg false discovery rate. Cronbach α was calculated at baseline and end point for outcomes.

**Conclusions:**

The innovative 7-session HUS-iCBT for SAD (vs ≥9 sessions in most previous programs) may improve adherence without compromising effectiveness. Task shifting from highly specialized to less specialized professionals may further improve cost-effectiveness, although formal health-economic evaluation is needed. These findings may inform the development of future iCBT programs for mental disorders.

## Introduction

Social anxiety disorder (SAD), or social phobia, is a common mental health condition with an annual prevalence of around 2.4% [1]. Persistent, irrational fear of social situations in SAD can lead to poor academic performance [2], affect interpersonal relationships [3], and lower quality of life [4]. SAD is often accompanied by depression and other anxiety disorders [5], and some patients appear to misuse alcohol in an effort to control the symptoms of SAD [6]. The first-line treatments for SAD are pharmacotherapy and cognitive behavioral therapy (CBT) [7,8]. CBT is the most extensively studied and efficacious psychotherapy for patients with SAD, showing an effect size of *d*=0.9 to 1.2 [9]. The availability of conventional, face-to-face CBT is, however, limited and does not meet the increasing demand. Digital interventions, such as guided internet-delivered cognitive behavioral therapies (iCBTs), demonstrate comparable efficacy in randomized controlled trials (RCTs) but offer enhanced accessibility, affordability, and hence, scalability [10]. This also applies to iCBT for SAD [11-14].

Although RCTs are the gold standard for evidence, their results are not always confirmed in routine clinical care. Therefore, real-world effectiveness studies are also needed [15,16]. To the best of our knowledge, only a few such studies have been published on therapist-guided iCBT for SAD in adults [17-20]. They comprised different sample sizes (most often small) and employed different methodologies and various measures. Interestingly, one of these studies comprised 6 sessions (“modules”) [20] yet yielded better adherence and symptom reduction comparable to those of studies comprising 9 [18] or 12 [17] sessions (one of the studies did not report the number of sessions). Moreover, a recent study [21] demonstrated that in some somatic conditions, iCBT programs with fewer modules were more effective in reducing anxiety symptoms.

Helsinki University Hospital (HUS) has developed and provides several original nationwide iCBT programs (hereafter referred to as HUS-iCBTs) for mental disorders that have demonstrated encouraging efficacy for depression [22] and effectiveness for insomnia [23] and generalized anxiety disorder [24]. HUS-iCBTs also include an innovative program for SAD. Compared with existing interventions, this program is designed to reduce both patient burden and provider resource requirements. First, it consists of 7 sessions, compared with the more common treatment formats of 9 or more sessions. Second, the specialized university hospital clinic delivering the intervention has optimized resource allocation by delegating screening interviews to well-instructed referring physicians and by restricting the target population to individuals with clinically significant SAD, rather than to those with subclinical symptoms.

The current observational study was set up to establish the effectiveness of this program in routine clinical care and to evaluate the advantages and disadvantages of the methodology used. We aimed to estimate (1) the symptom reduction rate during iCBT, expecting it to match that of the previously developed programs. Furthermore, we presumed that (2) due to its smaller number of sessions, our program could enhance adherence.

## Methods

### Design and Setting

This is a retrospective, observational, register-based cohort study. HUS’s Department of Psychiatry has developed and is providing nationwide, internet-based, therapist-guided iCBT programs in Finnish and Swedish languages for several psychiatric conditions (HUS-iCBTs), including the one for SAD. These flexible time-scheduled programs require a physician’s referral and are reimbursed by the public sector, meaning they are free for patients. Therapist guidance is administered centrally by a specialized university hospital clinic.

Predictors in our model for change in SPIN scores across treatment (primary outcome) included age, gender, municipality (urban or rural), Patient Health Questionnaire-9 (PHQ-9) and Overall Anxiety Severity and Impairment Scale (OASIS) baseline scores, completed session count, end point status (dropout, defined as discontinuation before session 7, or completer, defined as the completion of all 7 sessions), time spent in therapy, and the interactions between these variables.

### Inclusion Criteria for HUS-iCBT Program for SAD

To be accepted for the SAD program, the patients need to have SAD diagnosis. To keep our program as minimally resource-consuming as possible, HUS does not perform a diagnostic screening interview before starting the program. Instead, HUS relies on the diagnosis made by the referring physicians, which is based on their clinical judgment supplemented by the mandatory verification of SAD diagnosis using criteria listed in the *ICD-10* (*International Classification of Diseases*, *Tenth Revision*) [25]. HUS does not provide additional guidelines for establishing the diagnosis. Patients must have an email address and official e-identification. Severe personality disorders, suicidal behavior, acute psychosis or mania, and neurological or neuropsychiatric disorders with cognitive decline are exclusion criteria.

### Inclusion Criteria for the Current Study

All patients who entered HUS-iCBT for SAD between 2019 and 2022 were included in this registry study if they met the additional severity criterion of a baseline SPIN score of ≥21—a score that appears to reliably distinguish clinical disorder from subclinical symptoms [26,27]. Only patients with at least one on-treatment session (session 2) were included in the modified intent-to-treat (mITT) population, as session 1 consisted only of an introduction to SAD and HUS-iCBT (ie, treatment was not started). Those aged <18 years, with missing baseline information, who did not log-in, or who reentered the treatment were excluded. The population was identified based on a feasibility analysis, with no formal power calculations.

### Ethical Considerations

The study followed ICH-GCP (International Council for Harmonisation–Good Clinical Practice) guidelines, European legislation for personal data safety, General Data Protection Regulation (GDPR), and Finland’s regulations. It was approved by the pertinent institutional authorities (HUS/11850/2022). As a registry study, it did not require approval from the ethics committee or require informed consent. The data are stored in a protected HUS “Acamedic” environment. Only researchers who had appropriate permits, credentials, and signed a confidentiality and data security commitment could access the data. All personal data were treated as confidential and were pseudonymized, preventing researchers from reidentifying individual patients. No identification of individual patients in any images in the paper or supplementary materials is possible. Patients were not compensated for participation in the study.

### Assessments

#### Overview

Web-based questionnaires were used to assess symptoms. The primary measure of the severity of SAD was the Social Phobia Inventory (SPIN) [28]; secondary measures were the OASIS [29] for overall anxiety and anxiety-related impairment and the PHQ-9 [30] for the severity of depressive symptoms. Patients completed SPIN and PHQ-9 at first, third, fifth, and seventh sessions. OASIS was filled at the first, third, and seventh sessions.

#### Primary Outcome Measures

The symptom reduction rate was measured using the SPIN—a self-rating tool consisting of 17 items designed to assess the severity of SAD. Participants rate their experiences over the past week, with items covering various symptom domains of SAD, including fear, avoidance, and physiological arousal. The SPIN score is determined by summing the individual item scores [28]. In our study, the Cronbach α reliability was .88 at baseline and .90 at the end point.

Adherence was defined as the average number of completed sessions. Patients who completed all 7 sessions were categorized as completers. Those who finished less than 7 sessions were further categorized into early dropouts (completed <5 sessions) or late dropouts (completed 5‐6 sessions).

#### Secondary Outcome Measures

OASIS offers a brief, continuous self-assessment of anxiety’s overall severity and anxiety-related impairment across anxiety disorders. It consists of 5 questions, where participants rate their experiences over the past week on a scale from 0 to 4. The final score can range from 0 to 20. An 8-point cutoff accurately identifies the presence or absence of an anxiety disorder diagnosis in 87% of a clinical population sample [29]. In this study, the Cronbach α reliability was .85 at baseline and .88 at the end of the treatment.

The PHQ-9 is a widely used self-rating tool for screening, diagnosing, monitoring, and assessing the severity of depression [30]. It is based on the 9 diagnostic criteria for major depressive disorder as defined in the *Diagnostic and Statistical Manual of Mental Disorders*, *Fourth Edition* (*DSM-IV*) [31]. Internal consistency has ranged from 0.86 to 0.89 in 2 large clinical samples. Previous studies have shown that individuals with a high score (≥10) on the PHQ-9 were 7 to 13.6 times more likely to receive a depression diagnosis [30]. In the current study, the Cronbach α reliability was .91 at baseline and .92 at the end of the treatment.

### Intervention and Procedure

The HUS-iCBT for SAD program is primarily based on the cognitive model of social anxiety proposed by Clark and Wells [32]. It consists of 7 consecutive sessions, which include a combination of textual content, video materials, educational illustrations, case-based examples, therapeutic exercises, and homework assignments that focus on different areas of SAD (Textbox 1). Upon approval, participants receive an invitation to start the program. A structured schedule of one session per week is recommended, but participants can create their own schedule provided that a minimum interval of 24 hours is maintained between sessions. There is no limit on completion time, as long as the participant remains engaged in the program. The initial 4 sessions of the program primarily focus on theoretical insights, while the subsequent sessions introduce more practical elements and supplementary content (Textbox 1).

Textbox 1. **Content of sessions 1‐7.**

Session 1: Introduction to HUS-iCBT. Rationale of iCBT treatment. Information on SAD.

Session 2: Facing your fears. Safety behaviors. Automatic thoughts.

Session 3: Beliefs and interpretations. Effect of attention to fears. Chain analysis of responding to fear. Building an exposure hierarchy.

Session 4: Common cognitive biases. Thought diary. Fears related to exposure. Starting in vivo exposure.

Session 5: Assessing exposure practice. Relaxation training. Continuing in vivo exposure.

Session 6: Assessing exposure practice. Fear-maintaining factors. Focusing attention. Reformulation. Continuing in vivo exposure.

Session 7: Assessing exposure practice. Exposure as a philosophy. Social skills. Long-term plan.

In the beginning, a short kick-off call (instead of a comprehensive screening interview) is conducted, where the therapist attempts to reach the patient. The intention is to facilitate alliance building, ensuring that the patient correctly understands the nature of guided iCBT and knows how to log in. If the patient does not answer the call, a welcoming text message is sent along with the log-in instructions.

During therapy, patients are contacted approximately once per week with written messages on the platform, text messages, or phone calls. Throughout the treatment, a therapist monitors progress, gives personalized feedback, provides encouragement, and reinforces therapeutic strategies. After 2 weeks of inactivity (or 2 missed sessions, if the patient follows the recommended schedule of one session per week), the therapist calls or sends a text message inquiry. If the patient does not continue for a month, the treatment is discontinued. Before discontinuation, at least one call is attempted. Although time spent by the therapist on the specific treatment is not measured, a quota of patients per working hour is maintained. This would, on average, be roughly 10 minutes of working time per patient per week.

Suicide risk is assessed by therapists using multiple markers, including symptom severity (eg, an elevated score in PHQ-9 scale item 9), patient responses, complaints, and other risk factors, such as alcohol misuse. Additional questions about suicidal ideation or intent are also asked during therapy. If any of these markers suggest a heightened risk of suicide, the therapist attempts to call the patient; if the patient cannot be reached, a follow-up text message is sent. If no response is received within 2 days, the referring physician is notified by telephone.

The background, training, and supervision of HUS-iCBT therapists have been described in Ritola et al [24]. However, the authors described an earlier version of the HUS-iCBT therapist support protocol, which has since been updated to include more contact. In this patient cohort, the new protocol was used exclusively (see *Intervention and Procedure* section*)*.

### Statistical Analysis

The significance level was set at .05 for the main tests and .01 for post hoc tests. Covariance structures were compared, including homogeneous, diagonal (heterogeneous variances), autoregressive heterogeneous (ARH1), and unstructured models using the maximum likelihood (ML) method. The unstructured model did not converge and was excluded. The ARH1 model, which accounts for changing variances and declining correlations between time points, provided the best fit according to Akaike and Bayesian information criteria (AIC/BIC) and likelihood ratio tests. Final parameter estimates were obtained using the restricted maximum likelihood (REML) method with the ARH1 structure. Exploratory analyses were conducted using predictors available from the registry, such as sex, age, baseline scores, municipality, and time spent in treatment. Each of these was included to observe whether it could interact with the SPIN outcomes ().

Independent-samples *t* test and chi-square test were used for between-group comparisons. Effect sizes were calculated using last observation carried forward (LOCF) values with Cohen *d* interpretations based on conventional guidelines: small (*d*=0.2), medium (*d*=0.5), and large (*d*=0.8). For the mITT population, we calculated Cohen *d* for SPIN using a linear mixed effects model with REML estimation.

We calculated reliable change using LOCF values, following the formula established by Jacobson and Truax [33]. The thresholds for determining reliable improvement or deterioration varied for each measure.

The Little MCAR (missing completely at random) test was used for missing data analysis, and the Benjamini-Hochberg false discovery rate correction was employed to compensate for multiple testing. The primary linear mixed effects model was repeated as a sensitivity analysis using 50 imputed datasets . Cohen *d* was estimated from pooled marginal mean change. Cronbach α was calculated at baseline and at the end of treatment to determine the internal consistency of the primary and secondary outcomes. We used IBM SPSS Statistics version 29 for the analyses.

## Results

### Descriptives

**Figure 1.**
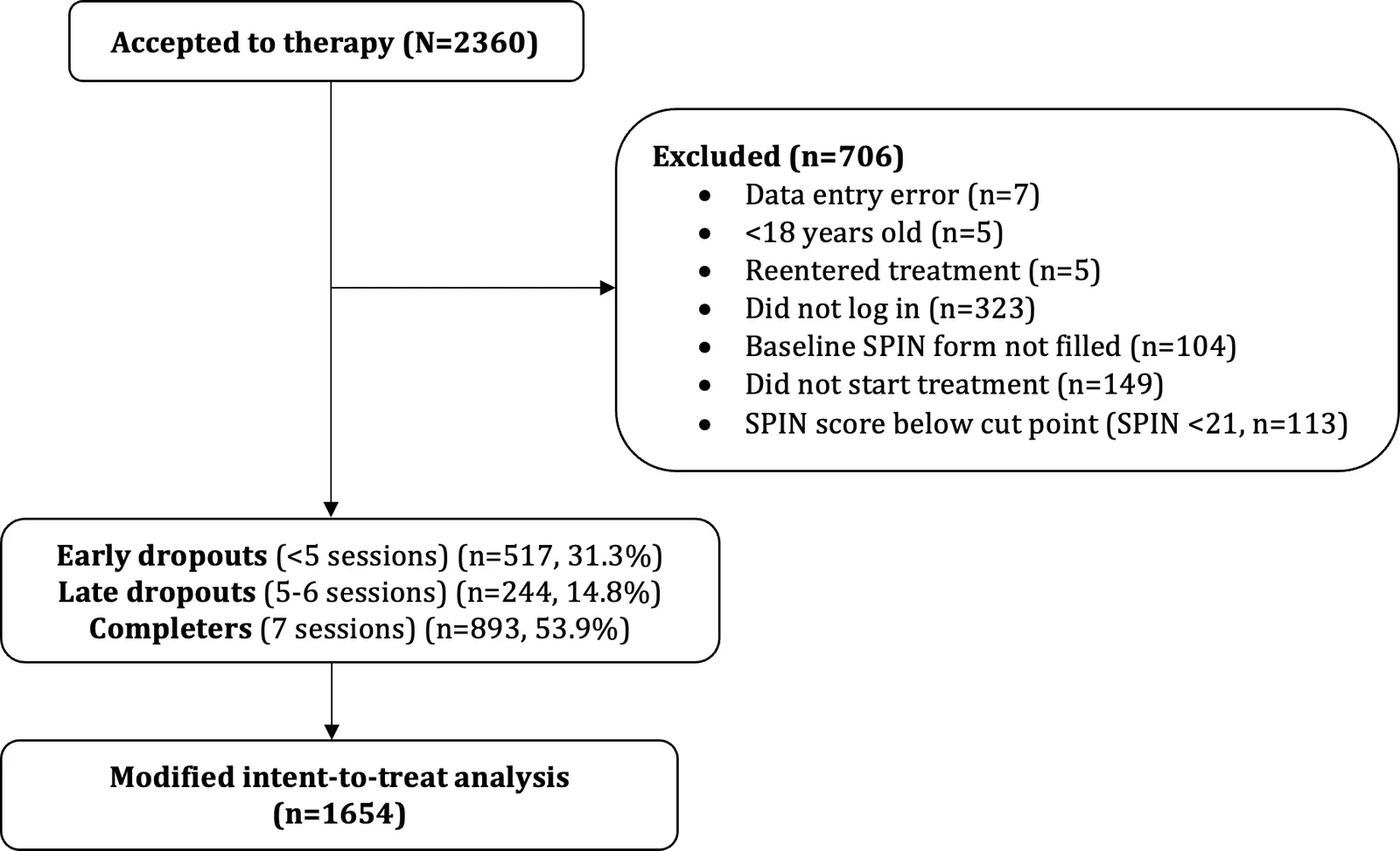
Patient flowchart. Patients with ≥1 on-treatment sessions (session 2) formed the modified intent-to-treat population. “Did not start treatment”—patients who logged in but did not proceed to session 2, where treatment was started. SPIN: Social Phobia Inventory.

The participants were 1654 adults from different parts of Finland who were diagnosed with SAD (Figure 1). Most participants came from an urban municipality (n=1473, 89.5%; Table 1). At baseline, statistically significant between-group differences were observed for age (the completers were 1.7 years older than noncompleters), sex (more females completed the treatment than males), PHQ-9 scores (lower by 2 points in the completers), and OASIS scores (lower by 1 point in the completers population) but not for SPIN (Table 1).

**Table 1.** Population description and baseline score comparison.

Characteristics	Total sample, n/N (%)	Completers, n/N (%)	Noncompleters, n/N (%)	Completers vs noncompleters test statistic (χ^2^/*t* test) (*df*)	*P* value
Sex				9.6 (1)^a^	.002
Female	1026/1654 (62)	585/893 (65.5)	441/761 (58)		
Male	628/1654 (38)	308/893 (34.5)	320/761 (42)		
Municipality^b^				0.6 (1)^a^	.44
Urban	1473/1646 (89.5)	789/887 (89)	684/759 (90.1)		
Nonurban	173/1646 (10.5)	98/887 (11)	75/759 (9.9)		
Age (y), mean (SD)	30.5 (10.2)	31.3 (11)	29.6 (9)	−3.632 (1649)^c^	<.001
SPIN^d^, mean (SD)	42.6 (10.4)	42.3 (10.4)	42.9 (10.4)	1.240 (1652)^c^	.11
OASIS^e^, mean (SD)	11.8 (3.7)	11.3 (3.7)	12.3 (3.6)	5.229 (1652)^c^	<.001
PHQ-9^f^, mean (SD)	9.5 (5.9)	8.6 (5.7)	10.6 (6)	6.787 (1574)^c^	<.001

a Chi-square test.

b Participants who had provided their municipality.

c *t* test.

d SPIN: The Social Phobia Inventory-17-item self-rating for social anxiety disorder.

e OASIS: Overall Anxiety Severity and Impairment Scale.

f PHQ-9: Patient Health Questionnaire-9.

### Primary Outcome Model

As shown in Table 2, on average, patients improved by 2.2 (95% CI 1.49‐3.0) points every other session (evaluated at sessions 1, 3, 5, and 7). Patients who exhibited higher PHQ-9 scores at baseline tended to maintain higher scores on the SPIN scale throughout the treatment. Similarly, individuals with initially higher OASIS scores also tended to demonstrate higher scores on the SPIN, likely due to the broader assessment of anxiety provided by the OASIS. The baseline PHQ-9 score interacted directly with the by-session changes in the SPIN score over the course of treatment, indicating a linear effect. Male sex and status “dropout” were associated with lower predicted scores. After applying false discovery rate correction (excluding intercept), only 5 parameters remained statistically significant—OASIS and PHQ-9 baseline scores, interaction with PHQ-9 baseline score and session count, session count, and male sex. For more details, see Tables S1, S 2 and S3 in Multimedia Appendix 1.

**Table 2.** Predictions of changes in Social Phobia Inventory (SPIN) scores^a^.

Parameter	Estimate (SE; 95% CI)	*t* test (*df*)	*P* value
Intercept	32.09 (1.48; 29.20 to 34.99)	21.74 (1765.6)	<.001
OASIS^b^ baseline score	1.10 (0.08; 0.95 to 1.26)	14.14 (1721.2)	<.001
PHQ-9^c^ baseline score	0.36 (0.05; 0.26 to 0.45)	7.36 (1722.4)	<.001
PHQ-9 baseline score & session count^d^	0.05 (0.01; 0.03 to 0.07)	4.15 (1274.5)	<.001
Session count	−2.24 (0.38; −3.00 to −1.49)	−5.84 (1819.7)	<.001
Male sex	−3.07 (0.49; −4.03 to −2.12)	−6.30 (1722.2)	<.001
Status: dropout	−1.08 (0.49; −2.04 to −0.12)	−2.20 (1730.0)	.03
Time spent in therapy & session count^d^	−0.01 (0.004; −0.01 to 0.001)	−1.81 (3015.4)	.07
Municipality: urban	−1.58 (0.95; −3.44 to 0.28)	−1.67 (1718.1)	.10
Male sex & session count^d^	0.18 (0.11; −0.04 to 0.40)	1.61 (1252.9)	.11
Age	−0.04 (0.02; −0.08 to 0.01)	−1.56 (1723.2)	.12
Status: dropout session count^d^	0.14 (0.15; −0.15 to 0.43)	0.94 (1987.1)	.35
OASIS baseline score & session count^d^	0.01 (0.02; −0.02 to 0.05)	0.72 (1240.8)	.47
Age & session count^d^	−0.003 (0.01; −0.01 to 0.01)	−0.54 (1145.1)	.59
Time spent in therapy	−0.002 (0.02; −0.04 to 0.03)	−0.13 (3282.2)	.90

a Mixed linear model parameter estimates.

b OASIS: Overall Anxiety Severity and Impairment Scale.

c PHQ-9: Patient Health Questionnaire-9.

d Symbolizes the interaction of 2 parameters.

### Observed Outcomes

#### Dimensional Outcomes

**Figure 2.**
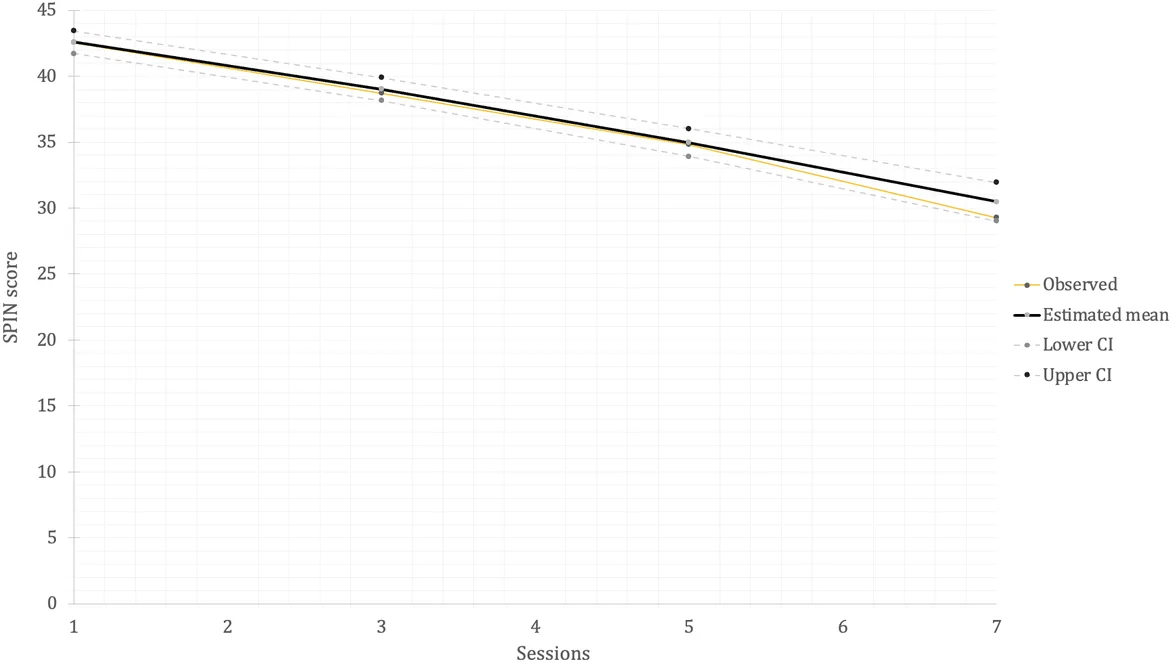
Estimated mean marginal values based on a linear mixed model vs observed values. The curves represent the typical path of a patient’s progress over time. SPIN: Social Phobia Inventory.

The SPIN-measured effect size was large for mITT (*d*=1.09, 95% CI 1.03‐1.16) and for completer (*d*=1.15, 95% CI 1.06‐1.23) populations, medium for late dropouts (*d*=0.72, 95% CI 0.58‐0.86), and low for early dropouts (*d*=0.41, 95% CI 0.32‐0.50). Moreover, completers demonstrated a medium effect size on both OASIS and PHQ-9 (Table 3). The model-estimated mean trajectory is shown in Figure 2.

**Table 3.** Changes in outcome measures across different populations^a^.

Treatment effect	Baseline, mean (SD)	Post, mean (SD)	Pre-post, change	Pearson *R*	Effect size, Cohen *d*^b^ (95% CI)
SPIN^c^					
Completers	42.3 (10.4)	29.6 (14)	− 12.7	0.623	1.15 (1.06‐1.23)
Early dropouts	43.1 (10.6)	40.2 (12.1)	− 2.9	0.810	0.41 (0.32‐0.50)
Late dropouts	42.4 (9.9)	35.4 (13.3)	− 7.0	0.669	0.72 (0.58‐0.86)
OASIS^d^					
Completers	11.3 (3.6)	8.6 (4.3)	− 2.7	0.599	0.77 (0.69‐0.84)
PHQ-9^e^					
Completers	8.6 (5.7)	6.8 (6.0)	− 1.8	0.737	0.46 (0.39‐0.52)

a Values were estimated using the last observation carried forward (LOCF) method.

b Cohen *d* effect size: small=0.2; medium=0.5; large=0.8.

c SPIN: Social Phobia Inventory.

d OASIS: The Overall Anxiety Severity and Impairment Scale.

e PHQ-9: Patient Health Questionnaire-9.

For SPIN, our calculations indicated that a change of ≥10 points was reliable. For the mITT population, reliable improvement was demonstrated in 41.1% of patients, while reliable deterioration was observed in only 1.7% of patients (Table 4). Completers demonstrated even more favorable results, where 57.8% (516/893) improved reliably and 30.2% (270/893, 95% CI 27.3‐33.3) achieved the subclinical range on SPIN (score of <21) [26,27]. In the mITT population, the subclinical range was reached by 329 out of 1654 (19.9%, 95% CI 18.0‐21.9) patients, in early dropouts by 26 out of 517 (5%, 95% CI 3.5‐7.3) patients, and in late dropouts by 33 out of 244 (13.5%, 95% CI 9.8‐18.4) patients. Reliable changes were also observed in secondary outcomes, including OASIS (≥4 points) and PHQ-9 (≥7 points). On OASIS, 26.2% (433/1654) of patients showed reliable improvement and 3.1% (51/1654) showed reliable deterioration, with corresponding figures of 8.5% (141/1654) and 3.0% (50/1654) for PHQ-9.

**Table 4.** Reliable changes from baseline to end point^a^.

Clinical index	mITT^b^ (N=1654), n (%)	Completers (n=893), n (%)	Late dropouts (n=244), n (%)	Early dropouts (n=517), n (%)
SPIN^c^				
Reliable improvement^d^	679 (41.1)	516 (57.8)	87 (35.7)	76 (14.7)
Reliable deterioration^e^	28 (1.7)	10 (1.1)	6 (2.5)	12 (2.3)
OASIS^f^				
Reliable improvement^g^	433 (26.2)	337 (37.7)	29 (11.9)	67 (13)
Reliable deterioration^h^	51 (3.1)	27 (3)	11 (4.5)	13 (2.5)
PHQ-9^i^				
Reliable improvement^j^	141 (8.5)	93 (10.4)	25 (10.2)	23 (4.4)
Reliable deterioration^k^	50 (3.0)	28 (3.1)	6 (2.5)	16 (3.1)

a Values were calculated using Reliable Change Index (RCI) with the last observation carried forward method.

b mITT: intent-to-treat population.

c SPIN: Social Phobia Inventory.

d Reliable improvement—SPIN score drop of ≥10 points.

e Reliable deterioration—SPIN score increase of ≥10 points.

f OASIS: Overall Anxiety Severity and Impairment Scale.

g An OASIS score drop of ≥4 points.

h An OASIS score increase of ≥4 points.

i PHQ-9: Patient Health Questionnaire-9.

j Reliable improvement—PHQ-9 score drop of ≥7 points.

k Reliable deterioration—PHQ-9 score increase of ≥7 points.

#### Categorical Outcomes

SPIN score <19 (a score often used to distinguish patients with SAD from healthy controls [28]) was achieved in 271 out of 1654 (16.5%, 95% CI 14.6‐18.2) patients in the mITT population. The corresponding proportions among early dropouts, late dropouts, and completers were 22 out of 517 (4.3%, 95% CI 2.8‐6.4), 28 out of 244 (11.5%, 95% CI 8.0‐16.2), and 223 out of 893 (25%, 95% CI 22.3‐27.9).

At baseline, suicidal thoughts (usually rare) were present more often in dropouts (especially in early dropouts) than in completers. They decreased in all subgroups with statistical significance (Table S4 in Multimedia Appendix 1).

On average, patients completed 5.74 (SD 1.99) of the 7 sessions (82%). More than half of all patients (893/1654, 54%) completed the HUS-iCBT program. The average time spent in therapy was 86 (SD 30, range 9‐589) days for the entire sample, 83 (SD 25, range 9‐190) days for early dropouts, and 105 (SD 24, range 49‐250) days for late dropouts. Completers spent an average of 82 (SD 33) days in therapy, and the completion time ranged from 15 to 589 days. Time spent in treatment was explored as a potential predictor of change in SPIN scores in the primary outcome model; however, no statistically significant effect was observed (Table 2).

#### Missing Data

The Little MCAR test was not statistically significant for SPIN scores alone (*χ*²_6_=9.40, *P*=.15) but became significant when combined with PHQ-9 and OASIS scores (*χ*²_42_=98.71, *P*<.001), indicating that missingness was not completely at random. These findings were expected, as patients who discontinued treatment did not provide responses in subsequent sessions, contributing to missing data (Table S5 in Multimedia Appendix 1). Additionally, some missing data resulted from participants attending sessions without completing the outcome questionnaires. In some cases, participants were categorized as “completers” despite their last available data originating from session 5 rather than session 7. The primary mixed linear model included all available repeated outcome measures and covariates (eg, age, gender, municipality, and treatment length), and the results were interpreted under the missing-at-random assumption.

In addition to our original mixed linear model, we repeated the analysis using 50 multiply imputed datasets (Table S6 in Multimedia Appendix 1). The pooled estimated marginal mean for SPIN decreased from 42.91 (95% CI 42.10‐43.73) at baseline to 30.33 (95% CI 29.03‐31.62), which is generally consistent with the primary analysis. An estimated effect size of *d*=1.21 (95% CI 1.15‐1.27) was obtained, which is slightly larger but still comparable to the previously reported *d*=1.09 (95% CI 1.03‐1.16).

## Discussion

### Principal Findings

In this retrospective, observational, register-based cohort study, we examined the effectiveness of our original, nationwide-delivered, therapist-guided, flexible time-schedule, free (for patients), 7-session HUS-iCBT program for SAD in a large sample (N=1654). We observed a large effect size on the primary outcome measure (SPIN) for both mITT and completer populations (*d*=1.09 and *d*=1.15, respectively). Both statistically and clinically significant improvements were also noted in the secondary outcomes: the severity of overall anxiety and anxiety-related impairment and of depression. In our mITT population, 41.1% (679/1654) of participants showed reliable SPIN-measured improvement, with even higher rates of 57.8% (516/893) for completers. Completers demonstrated more favorable outcomes than late and, in particular, early dropouts. Reliable deterioration rates were low among all groups. Our patients also demonstrated a favorable adherence rate: the average completion rate was 5.74 (SD 1.99) of altogether 7 sessions, resulting in an overall session completion rate of 82%. More than half of the patients (893/1654, 54%) completed the program.

### Comparison With Prior Work

In all 4 earlier published observational studies the authors were able to locate, iCBT was effective for SAD. El Alaoui et al [17] (N=654) demonstrated an effect size of *d*=0.86 using the Liebowitz Social Anxiety Scale–Self-Rated (LSAS-SR). Nordgreen et al [18] (N=169) also reported large effect sizes *d*=1.00 on the Social Phobia Scale and *d*=1.10 on the Social Interaction Anxiety Scale. Likewise, favorable results were demonstrated by Dryman et al [19] (N=3384) using SPIN, where effect sizes ranged from 0.63 to 1.40, depending on completed parts of treatment. Significant improvement was also observed in secondary measures. Williams et al [20] (Study 1: N=368; Study 2: N=192) reported large effect sizes across 2 pathways followed (*d*=0.82‐1.09) for the primary outcome using the Mini-SPIN and the Kessler-10 Psychological Distress Scale. Small effect sizes (*d*=0.36‐0.46) were observed for secondary outcomes using the World Health Organization Disability Assessment Schedule-II and PHQ-9.

Moreover, Nordgreen et al [18] demonstrated a reliable improvement in 66.2% of participants, while a reliable deterioration was observed in 16.6% of their participants. Dryman et al [19] evaluated the reliable change index only for “fully adherent” participants and reported that 72.3% of them improved reliably. El Alaoui et al [17] and Williams et al [20] did not report these measures.

All studies (including ours) defined adherence as an average of completed sessions/modules. Accordingly, El Alaoui et al [17] reported an average of 8.12 out of 12 (68%) sessions, while the patients in the study of Nordgreen et al [18] accomplished 5.3 out of 9 (59%) sessions (compared with 5.74 out of 7, 82% in our study). Dryman et al [19] reported an average of 12.14 of 14 “activities” and 1.53 of 13 “exposures” completed—a way of data presentation that makes comparisons with other studies difficult. Patients in Williams et al [20] completed 85.3% of sessions, which is comparable to our findings. Notably, their program was also short (consisting of 6 sessions of which, on average, 5.12 were completed) and yielded effect sizes approximately as large as those demonstrated in other studies. This supports our hypothesis that a program with a smaller number of sessions (6 or 7 sessions) may be as effective as most of the currently available ones with a greater number of sessions (9 or 12 sessions), while also improving adherence.

El Alaoui et al [17] reported remission rates of 20% using an established cutoff score for LSAS-SR (≤30). Other authors have not reported remission rates. To the best of our knowledge, no SPIN cutoff score has been used to define remission in SAD. Nevertheless, a SPIN score <19 was achieved by 271 out of 1654 (16.5%, 95% CI 14.6‐18.2) of our mITT population and by 223 out of 893 (25%, 95% CI 22.3‐27.9) of completers. This makes our findings seemingly comparable to those in that previous study. However, such comparison requires some caution.

A direct comparison of the results would be challenging overall, since all listed studies explored different populations and applied various methodologies. We used iCBT in a nationwide setting with physician-referred patients, while in the study carried out by El Alaoui et al [17], patients could be either self-referred or referred by a physician, whereas those of Nordgreen et al [18] were referred by their general practitioners, and Dryman et al [19] recruited their patients using a variety of unpaid and paid sources, including advertisements, social media, word of mouth, and therapist referrals. Williams et al [20] examined 2 care pathways. In the first pathway, patients were referred by a registered practitioner, who also supervised their treatment in the community. In the second pathway, practitioners were responsible solely for referrals, whereas assessments and supervision were undertaken by specialist clinicians. In our study, we relied on the diagnosis made by the referring physicians plus an additional cutoff SPIN-measured severity score for inclusion. In contrast, the groups led by El Alaoui et al [17], Nordgreen et al [18], and Williams et al [20] in the second pathway conducted face-to-face diagnostic interviews at screening. The latter might require more personnel resources than ours, but we were unable to test this assumption due to the lack of comparable pertinent data.

Also, completion time varied across studies from fixed (as in the studies by Nordgreen et al [18], El Alaoui et al [17], and Williams et al [20]) to flexible (as in ours and in that by Dryman et al [19]). The number and content of sessions also differed (see above).

Our treatment was provided free of charge to patients, whereas in other studies, patients had to pay. For example, El Alaoui et al [17] reported that the cost could not exceed US $155 per year, and in the study by Dryman et al [19], patients were charged US $99 per month after a 7-day free trial. Nordgreen et al [18] and Williams et al [20] did not report whether their patients had to pay for treatment.

All the aforementioned differences—source of referral, face-to-face screening interview, time schedule, and fees—could affect the patients’ motivation and adherence. For instance, Hilvert-Bruce et al [34] highlighted that adding a one-time fee significantly improved adherence. A study conducted by Staples et al [35] suggests that self-referred patients achieve better results compared to those referred by primary care providers. Furthermore, differences in the primary outcome measures used (SPIN, Social Phobia Scale, Social Interaction Anxiety Scale, LSAS-SR, mini-SPIN, and Kessler-10 Psychological Distress Scale) might partly explain variations in the reported effectiveness rates.

To summarize, compared with earlier studies, our findings are encouraging, as similar results were achieved in a clearly clinical, physician-referred (not self-referred) population, with a potentially lower burden on patients and specialized services.

### Strengths and Limitations

One of the strengths of this study is that we included participants from all over the country, resulting in a large and representative sample, with 89.5% (1473/1654) of participants residing in urban areas (which is close to Finland’s national urbanization rate of 87.1%) [36]. To stabilize the quality of the treatment, the therapist support was administered centrally by specifically trained and regularly retrained professionals in a specialized clinic.

Although (1) offering care free of charge, (2) maintaining scheduling flexibility, (3) including only physician-referred (rather than self-referred) patients, and (4) restricting the sample to individuals with higher symptom severity (SPIN ≥21) may each hinder clinical improvement (Hilvert-Bruce et al [34]; Staples et al [35]), these characteristics may also represent strengths, given that our outcomes were comparable to those of other programs without such restrictions.

Beyond SAD-specific symptoms and the severity of depression, we have also used OASIS to measure overall anxiety and anxiety-related impairment. This was advantageous, since about 90% of patients with SAD are also diagnosed with depression and other anxiety disorders [37]. The utility of OASIS enables comparison of the effectiveness of iCBT across a range of anxiety disorders regardless of the disorder-specific measures used [38], as we did earlier [39].

Our program included fewer sessions than most of those reported previously, which—along with another, earlier-reported, low-session-number program—may have reduced the burden on patients and, thus, explain a higher average adherence rate than in other studies. Additionally, we did not conduct a diagnostic interview at screening, which decreased the burden on the provider’s resources and could thereby enhance the scalability of the treatment. This could also be viewed as a limitation. However, the accuracy of SAD diagnosis provided by the referring physicians was improved by adding an inclusion criterion of a SPIN score of ≥21 (instead of the commonly used ≥19 [28]). According to the scoring guide for interpretation, this score reliably indicates clinically significant symptoms of SAD and has shown good discriminative validity with high sensitivity and specificity [26,27].

The absence of a control group is a limitation, as it prevents the separation of the specific effects of the therapy from the changes due to, for example, the natural course of the disease. However, the ever-growing interest in real-world evidence as a supplement to RCTs has recently expanded to digital health companies and providers due to the ability of digital solutions to enhance user engagement [40]. This emphasizes the necessity of such observational, naturalistic studies for eHealth services, as well. A recent systematic review [41] reaffirmed the persistent lack of studies demonstrating the outcomes of implementing iCBT in real-world settings.

To lower the threshold for enrollment and, hence, enhance accessibility and increase engagement, our program required only a few exclusion criteria. It imposed a low burden on patients—an approach that, despite these practical strengths, had certain limitations. For instance, we collected only minimal sociodemographic data and no data about concomitant treatments, which prevented an in-depth exploration of some plausible predictors and mediators of effectiveness and adherence.

From the point of view of value-based health care [42], the relationship between costs and treatment outcomes should be closely monitored and constantly evaluated for further development of treatments. However, we did not have comparative data on expenditures (consisting here chiefly of personnel time per encounter and per treatment performed), which was another limitation.

In several analyses, we used the LOCF method. This can be seen as both a strength and a limitation. First, it allowed us to include data from patients who discontinued the treatment before session 7 and, thus, did not complete the remaining questionnaires. On the other hand, this approach may affect the treatment outcome results, which should therefore be interpreted with caution. It should be noted, however, that our primary outcome was computed using a linear mixed effects model with REML estimation, allowing the incorporation of all available observations for each participant without using the LOCF approach. Additionally, it enabled the estimation of the effect size for the mITT population.

### Implications for Future Research

As the effectiveness of iCBT for SAD in the real world becomes evident, future development should focus, beyond improved effectiveness, on minimizing resource consumption, as well as on lowering the burden for patients, rather than on testing the effectiveness. In this respect, the evidence increasingly suggests that “less may be more.” In routine care, a reduced number of iCBT sessions for SAD may lower patient burden, thereby improving adherence without compromising effectiveness. This novel finding should inform future developments of iCBT for SAD and be examined in other clinical populations and disorders. Furthermore, our delegation of thorough screening to well-instructed referring physicians might optimize the use of service provider’s resources; however, pertinent health-economic research is warranted to test this hypothesis. In addition, the effects of time schedule (fixed or flexible), fees (vs free for patients), concomitant treatments, and socioeconomics should become a subject for further exploration.

### Conclusions

“Less may be more”: in contrast to the more common iCBT programs for SAD comprising ≥9 sessions, this innovative HUS-iCBT program consists of only 7 sessions. Nevertheless, in routine care, it appears to improve treatment adherence without compromising effectiveness. Moreover, reallocating tasks from highly specialized providers to appropriately instructed less specialized professionals may enhance the cost-effectiveness of iCBT, although this hypothesis warrants evaluation in future health-economic studies. These findings may inform the development of future iCBT programs for SAD and potentially for other mental health disorders.

## Supplementary material

10.2196/85676 Multimedia Appendix 1 Additional information on approach to outcome calculations, suicidality, and missing data.

## Abbreviations

AIC/BIC: Akaike and Bayesian information criteria
ARH1: autoregressive heterogeneous
CBT: cognitive behavioral therapy
*DSM-IV*: *Diagnostic and Statistical Manual of Mental Disorders*, *Fourth Edition*
GDPR: General Data Protection Regulation
HUS: Helsinki University Hospital
iCBT: internet-delivered cognitive behavioral therapy program
*ICD-10*: *International Classification of Diseases*, *Tenth Revision*
ICH-GCP: International Council for Harmonisation–Good Clinical Practice
LOCF: last observation carried forward
LSAS-SR: Liebowitz Social Anxiety Scale–Self-Report
MCAR: missing completely at random
mITT: modified intent-to-treat
ML: maximum likelihood
OASIS: Overall Anxiety Severity and Impairment Scale
PHQ-9: Patient Health Questionnaire-9
RCT: randomized controlled trial
REML: restricted maximum likelihood
SAD: social anxiety disorder
SPIN: Social Phobia Inventory
