# Supplementary material for: Internet-Delivered Cognitive Behavioral Therapy for Social Anxiety Disorder: Nationwide Retrospective Cohort Observational Study
Source: J Med Internet Res. 2026 Sep 10;28:e85676. doi: 10.2196/85676 (PMC13561402; doi:10.2196/85676)
Supplement: Supplementary file 1 [file jmir-v28-e85676-s001.docx]

Multimedia appendix.

Table 1 & 2 Comparison of three linear mixed-effects models with different residual structures: Homogeneous (compound symmetry), Diagonal (unstructured variance), and ARH(1) (heterogeneous autoregressive). Table 3. shows the estimates of covariance parameters.

Table 1.

| Model | Residual Structure | –2 Log Likelihood | AIC | BIC |
| --- | --- | --- | --- | --- |
| 0 | Homogeneous | 36351.9751 | 36389.9751 | 36514.4150 |
| 1 | Diagonal | 36227.4598 | 36271.4598 | 36415.5481 |
| 3 | ARH(1) | 35827.3987 | 35873.3987 | 36034.0364 |

AIC: Akaike information criterion

BIC: Bayesian information criterion

Table 2.

| Model | –2 LL | k | Δdf | Δ–2LL | *P* (χ²) | ΔAIC | ΔBIC |
| --- | --- | --- | --- | --- | --- | --- | --- |
| Homogeneous | 36351.9751 | 1 | – | – | – | – | – |
| Diagonal | 36227.4598 | 2 | 1 | 124.5153 | <.001 | 118.5153 | 98.8669 |
| ARH(1) | 35827.3987 | 3 | 2 | 524.5764 | <.001 | 516.5764 | 480.3786 |

-2LL: Minus 2 Log-Likelihood

k: Number of residual parameters estimated

Δdf : Difference in degrees of freedom versus baseline

Δ–2LL : Change in log-likelihood

P (χ²): P-value from chi-square test comparing model fit

ΔAIC / ΔBIC: Change in AIC or BIC relative to the baseline (Model 0)

Table 3.

| Parameter | Estimate | Std. Error | Wald Z | *P* value | 95% CI Lower Bound | 95% CI Upper Bound |
| --- | --- | --- | --- | --- | --- | --- |
| Session 1 | 58.634 | 6.831 | 8.584 | <.001 | 46.664 | 73.674 |
| Session 3 | 93.514 | 7.399 | 12.638 | <.001 | 80.081 | 109.201 |
| Session 5 | 97.278 | 8.461 | 11.497 | <.001 | 82.031 | 115.359 |
| Session 7 | 115.738 | 9.342 | 12.389 | <.001 | 98.803 | 135.576 |
| ARH1 rho | .676 | .027 | 24.805 | <.001 | 0.619 | .726 |
| Intercept Variance | 16.157 | 6.838 | 2.363 | .02 | 7.049 | 37.033 |

Table 4. Suicidal thoughts present at baseline and end point.

| Population | Baseline  n/N, (% ,95% CI) | End point  n/N, (% ,95% CI) | Change in percentage | *P* value |
| --- | --- | --- | --- | --- |
| mITT | 456/1654  (27.6%, 25.4 – 29.7) | 388/1654  (23.5%, 21.4 – 25.5) | - 4.1 | < .001 |
| Completers | 195/893  (21.8%, 19.1 – 24.6) | 163/893  (18.3%, 15.7 – 20.8) | - 3.5 | .019 |
| Late dropouts | 72/244  (29.5%, 23.8 – 35.2) | 51/244  (20.9%, 15.8 – 26.0) | - 8.6 | .002 |
| Early dropouts | 184/517  (35.6%, 31.4 - 39.7) | 168/517  (32.5%, 28.5 – 36.5) | - 3.1 | .011 |

Info: PHQ-9 item 9 analyisis using last observation carried forward (LOCF) values.

Table 5. Little’s MCAR full test results for repeated outcome measures.

| Variable | N | Mean | SD | Missing, n (%) |
| --- | --- | --- | --- | --- |
| SPIN session 1 score | 1654 | 42.56 | 10.40 | 0 (0.0) |
| SPIN session 3 score | 1528 | 38.72 | 12.22 | 126 (7.6) |
| SPIN session 5 score | 1137 | 34.81 | 13.09 | 517 (31.3) |
| SPIN session 7 score | 871 | 29.28 | 13.93 | 783 (47.3) |
| PHQ-9 session 1 score | 1654 | 9.54 | 5.92 | 0 (0.0) |
| PHQ-9 session 3 score | 1525 | 8.95 | 5.99 | 129 (7.8) |
| PHQ-9 session 5 score | 1136 | 7.86 | 6.00 | 518 (31.3) |
| PHQ-9 session 7 score | 870 | 6.74 | 5.98 | 784 (47.4) |
| OASIS session 1 score | 1654 | 11.75 | 3.66 | 0 (0.0) |
| OASIS session 3 score | 1523 | 10.76 | 3.89 | 131 (7.9) |
| OASIS session 7 score | 870 | 8.61 | 4.30 | 784 (47.4) |

Info: Little's MCAR test: χ²(42) = 98.711, P < .001.

OASIS was filled only at sessions 1, 3 and 7.

Table 6. Predictions of changes in SPIN scores using linear mixed effects model with multiply imputed data sets.

| Parameter | Estimate | Std. Error | *t* test | *P* value | 95% CI Lower | 95% CI Upper |
| --- | --- | --- | --- | --- | --- | --- |
| Intercept | 31.77 | 1.45 | 21.88 | <.001 | 28.93 | 34.62 |
| OASIS baseline score | 1.11 | 0.08 | 14.48 | <.001 | 0.96 | 1.26 |
| PHQ-9 baseline score | 0.36 | 0.05 | 7.66 | <.001 | 0.27 | 0.46 |
| Session count | -2.26 | 0.39 | -5.77 | <.001 | -3.03 | -1.49 |
| Male gender | -2.96 | 0.48 | -6.17 | <.001 | -3.90 | -2.02 |
| ^a^PHQ-9 baseline score Session count | 0.03 | 0.01 | 3.07 | .002 | 0.01 | 0.06 |
| ^a^Time spent in therapy  Session count | -0.01 | 0.004 | -2.10 | .04 | -0.02 | -0.001 |
| Municipality - Urban | -1.68 | 0.93 | -1.81 | .07 | -3.50 | 0.14 |
| Status - Dropout | -0.77 | 0.48 | -1.61 | .11 | -1.71 | 0.17 |
| ^a^Male gender  Session count | 0.15 | 0.11 | 1.33 | .18 | -0.07 | 0.38 |
| ^a^OASIS baseline score Session count | 0.02 | 0.02 | 1.31 | .19 | -0.01 | 0.06 |
| Age | -0.03 | 0.02 | -1.29 | .20 | -0.07 | 0.02 |
| ^a^Age  Session count | -0.004 | 0.01 | -0.86 | .39 | -0.02 | 0.01 |
| Time spent in therapy | 0.02 | 0.02 | 0.67 | .50 | -0.03 | 0.06 |
| ^a^Status - Dropout  Session count | -0.04 | 0.13 | -0.29 | .77 | -0.30 | 0.23 |
| ^a^Municipality  Session count | 0.03 | 0.21 | 0.14 | .89 | -0.39 | 0.45 |

^a^Symbolizes the interaction of two parameters

Info: After applying false discovery rate (FDR) correction (excluding Intercept), five parameters remained statistically significant – OASIS and PHQ-9 baseline scores, session count, male gender and an interaction between PHQ-9 baseline score and Session count.
